# CRISPR-mediated excision of HTLV-1 reduces proviral loads in PBMCs from HAM/TSP patients

**DOI:** 10.1007/s13365-026-01333-7

**Published:** 2026-08-29

**Authors:** Samuel Brancazio, Kamel Khalili, Steven Jacobson, Rafal Kaminski

**Affiliations:** 1 Center for Neurovirology and Gene Editing/Department of Microbiology, Immunology and Inflammation, Lewis Katz School of Medicine, Temple University, Philadelphia, PA 19140 USA; 2 Viral Immunology Section, Neuroimmunology Branch, Disorders and Stroke, National Institute of Neurological, National Institutes of Health, Bethesda, MD 20892 USA

**Keywords:** CRISPR-Cas9, HAM/TSP, HTLV-1, PBMCs, Proviral loads

## Abstract

CRISPR technology is emerging as a promising therapeutic approach for eliminating chronic viral infections, such as herpesviruses and HIV. Here, for the first time, we demonstrate in vitro that CRISPR can be used to excise the HTLV-1 genome and reduce proviral loads in PBMCs from HAM/TSP (HTLV-1-associated myelopathy/tropical spastic paraparesis) patients. Single treatment with CRISPR-RNP (ribonucleoprotein) complexes composed of two gRNAs targeting the HTLV-1 env gene and 3’LTR sequences resulted in excision of a 2613 bp segment of the proviral genome, spanning tax and HBZ genes, without detectable off-target activity. Furthermore, CRISPR treatment led to over 50% reduction in proviral loads 5 days post-electroporation. Our data indicate that CRISPR-Cas9 gene editing can be used as a therapeutic strategy to eliminate HTLV-1 DNA from infected cells and may serve as a platform for curing HAM/TSP.

**Supplementary Information:**

The online version contains supplementary material available at https://doi.org/10.1007/s13365-026-01333-7.

## Introduction

HTLV-1 is a human-tropic pathogenic retrovirus with approximately 10 million people worldwide living with the infection (WHO 2021). This estimate is likely underreported due to sampling selectivity and infrequency. HTLV-1 infection alone causes a 57% increase in adjusted all-cause mortality due to chronic inflammation and immune dysfunction. Additionally, patients can develop debilitating HTLV-1-associated pathologies such as adult T-cell leukemia/lymphoma (ATLL) and HTLV-1-associated myelopathy/tropical spastic paraparesis (HAM/TSP), both at an incidence rate of roughly 2% (Verdonck et al. 2007; Bangham 2018; Miura et al. 2022). Neither causal factors for the development of these pathologies nor the HTLV-1-specific treatment has yet been elucidated (Araujo et al. 2021; Miura et al. 2022), largely due to a lack of inclusion in research and public health agendas owing to its perception as a rare infection and the Global Southern endemicity of the virus (Cunha et al. 2023; Maertens et al. 2024).

HAM/TSP is a progressive neuroinflammatory degenerative disease of the spinal cord leading to chronic back pain, loss of control over bladder/bowel function and limb movement, and eventual loss of ability to walk without aid (Verdonck et al. 2007; Miura et al. 2022). HAM/TSP patients typically carry the virus asymptomatically for decades before beginning to develop HAM/TSP symptoms. Due to the low rate of disease progression and lack of understanding of causal factors of development of HAM/TSP, preventative treatments are not an option. However, therapeutic intervention upon symptom initiation could prevent patient progression of HAM/TSP to the point of severe symptom onset. Current understanding of the disease mechanism involves spinal cord infiltration of aberrantly activated HTLV-1-infected CD4 + T cells, followed by HTLV-1-specific CD8 + T cells. This infiltration and subsequent interaction between infected CD4 + and effector CD8 + T cells leads to an inflammatory bystander mechanism thought to cause the myelitis and spinal cord atrophy seen as hallmarks of disease progression (Izumo 2010; Nakamura 2023). Additionally, these cells release pro-inflammatory cytokines, such as IFNg, into the spinal cord milieu stimulating astrocytes to secrete CXCL10 further recruiting CXCR3 + immune cells creating a positive feedback loop (Ando et al. 2013). Notably, HAM/TSP patients have higher proviral loads (PVLs) compared to asymptomatic HTLV-1 carriers (Kubota et al. 1994; Nagai et al. 1998; Grassi et al. 2011). Higher PVLs are associated with increased levels of spontaneous lymphoproliferation, elevated HTLV-1 expression, and expanded numbers of activated HTLV-1 specific CD8 + T cells (Asquith et al. 2005; Enose-Akahata and Jacobson 2019). Furthermore, within the HAM/TSP patient population, there is a strong correlation between rate of disease progression and PVL (Matsuzaki et al. 2001; Meekings et al. 2008). Currently, there is no effective treatment for HAM/TSP. Anti-retroviral therapy, cytokines (IFN-α), corticosteroids, antioxidants, allograft bone marrow transplantation, and anti-CCR4 mAb have been trialed for patient treatment with limited success (Davies and Taylor 2023; Mashkani et al. 2023; Sato et al. 2024).

Since the persistence of the HTLV-1 reservoir is maintained through the proliferation of infected cells, rather than active viral replication, HTLV-1 infection is refractory to conventional antiretroviral therapy (Fernandez et al. 2024). Simultaneously, reliance on host cell polymerase with proofreading ability for replication results in genomic stability of HTLV-1 sequences and lower sequence variability than other human retroviruses (Gessain et al. 1992; Verdonck et al. 2007), such as HIV-1, making it an enticing target for a gene therapy approach involving genetic engineering (Panfil et al. 2020; Scott and Morris 2021; Wilkie and Panfil 2022; Domingues et al. 2024).

The use of the CRISPR platform for excision of the HTLV-1 proviral sequence is a promising yet under-investigated approach that warrants further examination. Here, we describe a dual-guide CRISPR-Cas9 approach that efficiently excises the HTLV-1 proviral sequence and reduces PVL in PBMCs from a cohort of ten HAM/TSP patients, indicating its therapeutic potential for HAM/TSP treatment.

## Results

### HAM/TSP patient-derived HTLV-1 sequences from our cohort differ from the ATK-1 reference genome

Our early attempts to edit the HTLV-1 genome using CRISPR gRNAs designed based on the ATK-1 isolate reference genome (GenBank: J02029.1) were unsuccessful (not shown). The ATK-1 isolate belongs to HTLV-1a-Jpn (subtype a (cosmopolitan), subgroup B (Japanese)). To verify viral sequences in our cohort of ten HAM/TSP patients (Supplementary Table 1), we performed PCR amplification of the env-3’LTR region of the HTLV-1 genome, followed by Sanger sequencing using primers spanning the amplicon. Since HTLV-1 classification is based on nucleotide diversity within the LTR region (Verdonck et al. 2007), we aligned 3' LTR sequences from our cohort with representative LTR sequences available in the public HTLV-1 molecular epidemiology database (Araujo et al. 2012). As shown in Supplementary Fig. 1, all our 3’LTR sequences clustered with HTLV-1a-TC (subtype a (cosmopolitan), subgroup A (Transcontinental)) LTR sequences except for one, HAM/TSP donor NIC1284, which clustered with HTLV-1a-WA (subtype a (cosmopolitan), subgroup C (West African)). This is expected since our cohort is made up of American patients, and consistent with previous studies, showing that the frequency of the Transcontinental subgroup in HAM/TSP patients is significantly higher than in asymptomatic carriers or patients with ATL (Adult T-cell Leukemia) (Furukawa et al. 2000; Nozuma et al. 2017; Katsuya et al. 2019).

### Single treatment of HAM/TSP PBMCs with RNP-CRISPR results in excision of proviral genomes without off-target editing

**Fig. 1:**
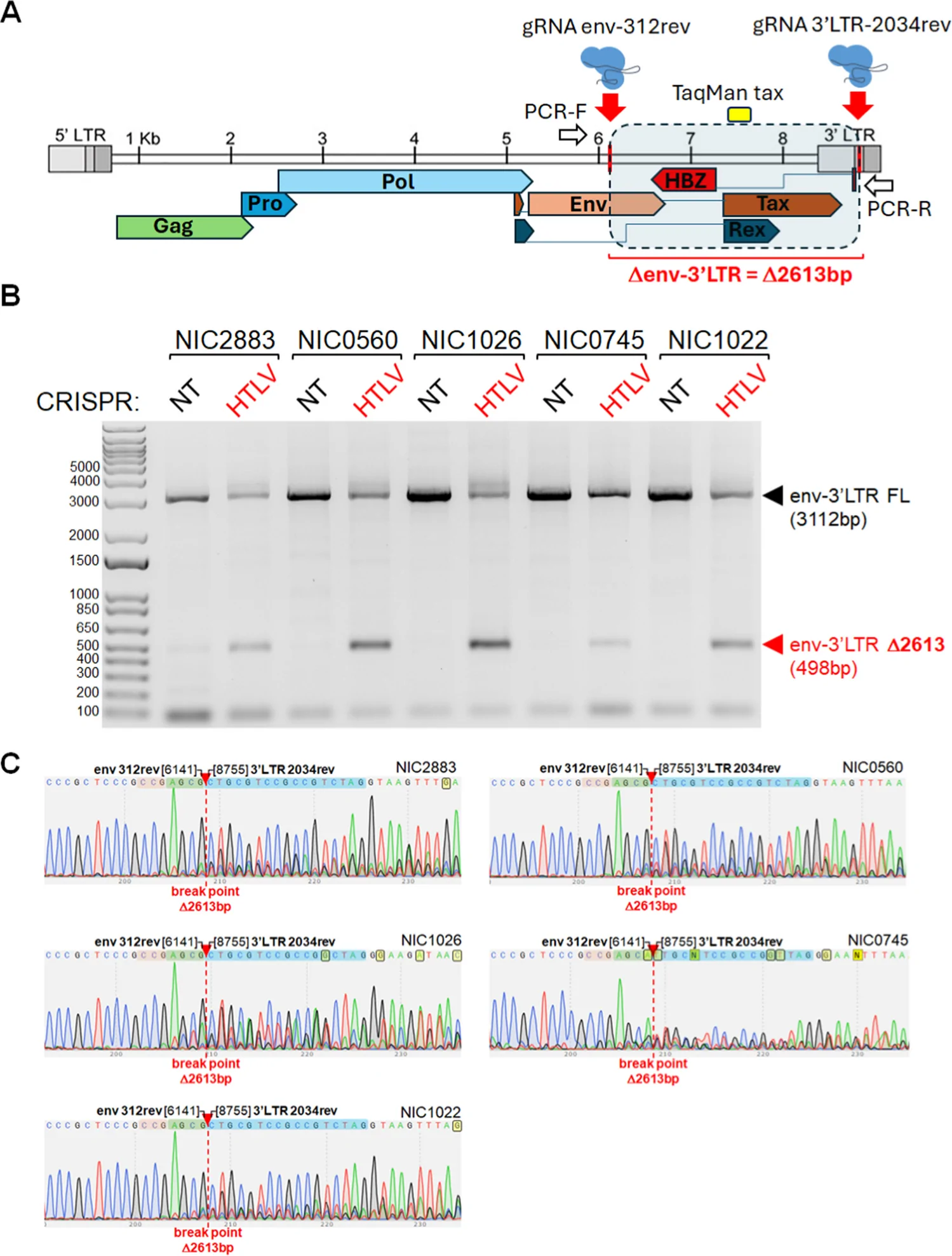
CRISPR-mediated excision of env-3’LTR region of HTLV-1 genome in PBMCs from HAM/TSP patients. **A** Schematic representation of the HTLV-1 genome with the location of major viral genes. Red arrows point to CRISPR gRNA target sites, white arrows depict PCR primer binding sites, and the yellow box shows the position of the HTLV-1 tax-specific TaqMan primer/probe set used for qPCR PVL estimation. Dashed, shaded box encompasses expected CRISPR-induced excision of env-3’LTR HTLV-1 region (Δ2613bp). **B** Agarose gel picture of PCR genotyping of HTLV-1 env-3’LTR region from five HAM/TSP patient PBMCs 2 days after treatment with non-targeting (NT) or HTLV-1-env + 3’LTR targeting (HTLV) Cas9/gRNA complexes. Black arrow points to 3112 bp full-length env-3’LTR amplicon (Suppl.Table 4., PCR primer set 2). Red arrow points to truncated 498 bp amplicon resulting from CRISPR-mediated excision of 2613 bp segment of HTLV-1 genome. **C** Sanger sequencing chromatograms of truncated amplicons from B). gRNA env-312rev target site is highlighted in green, 3’LTR-2034rev target site in blue, PAM sequences in red, and ambiguous/mismatched nucleotides in yellow. The site of CRISPR-excision “scars”, i.e., end-joined env-to-3’LTR sequences, are depicted by red arrows and a dashed red line (break point)

As our primary CRISPR-HTLV strategy, we selected excision of the HTLV-1 env-3' LTR region since this region spans, among others, the coding sequences of the tax and HBZ genes (Fig. 1A), which are critical for viral pathogenesis and the maintenance of the viral reservoir (Enose-Akahata et al. 2017). Based on the bioinformatic screen using donor NIC0071 sequence (HTLV-1a-TC) as a reference, we selected four of the top-scoring, measured by projected efficiency and specificity, candidate SpCas9 gRNAs that bind in the flanks of the env-3' LTR region (Supplementary Tables 2 and 3). Next, we tested them by electroporating ribonucleoprotein (RNP) complexes composed of recombinant SpCas9 and synthetic gRNAs (RNP-CRISPR) in PBMCs from the same donor (NIC0071). Based on PCR and InDel analyses, we selected two lead gRNAs, env-312rev and 3’LTR-2034rev, for further studies. CRISPR editing with these gRNAs resulted in 57% and 61% on-target InDel formation, respectively, when used separately, and verified excision of the 2613 bp segment of the HTLV-1 genome when used in combination (Supplementary Fig. 2). Subsequently, we used this gRNA pair for the remaining nine HAM/TSP donors. PBMCs were treated with control RNP-CRISPR-NT (SpCas9/non-targeting gRNA) or RNP-CRISPR-HTLV (SpCas9/env-312rev + 3’LTR-2034rev gRNAs). On days 2 and 5 post-CRISPR treatment, we collected cells for DNA analysis. Using single-round PCR, we detected a short 498-bp amplicon, corresponding to the CRISPR-cleaved/end-joined (Δ2613 bp) viral sequence, in CRISPR-HTLV-treated PBMCs from the five HAM/TSP donors having the highest PVLs in our cohort (NIC2883, NIC0560, NIC1026, NIC0745, and NIC1022) (Fig. 1B). Next, we verified excision of the env-3’LTR region by Sanger sequencing of 498-bp amplicons extracted from agarose gel (Fig. 1C). Additionally, we observed a clear reduction in the intensity of bands corresponding to full-length 3,112-bp amplicons in CRISPR-HTLV-treated cells relative to CRISPR-NT-treated cells (Fig. 1B). Since single-round PCR results were inconclusive for PBMCs from donors with lower PVLs (NIC0667, NIC3024, NIC1306, and NIC1284), we performed 2nd-round semi-nested PCRs and confirmed env-3’LTR in an additional three donors (NIC0667, NIC3024, and NIC1284) (Supplementary Fig. 3). Next, we performed off-target analysis. Because the HTLV-1 genome is highly divergent from the human genome, our two lead gRNAs have only a few nominated off-target sites: seven for env-312rev and eight for 3’LTR-2034rev, all with at least four mismatches relative to the target sequences (Supplementary Table 3). To experimentally verify the absence of off-target activity, we performed PCR amplification, followed by Sanger sequencing and InDel analysis, for all fifteen nominated off-target sites (seven for env-312rev and eight for 3’LTR-2034rev) (Supplementary Table 3 and 4) using genomic DNA from three successfully CRISPR-HTLV-treated donors (NIC0745, NIC1022, and NIC1026). As shown in Supplementary Figs. 4 and 5, we did not detect any InDel mutations at these sites.

### CRISPR-HTLV treatment reduces proviral loads

**Fig. 2:**
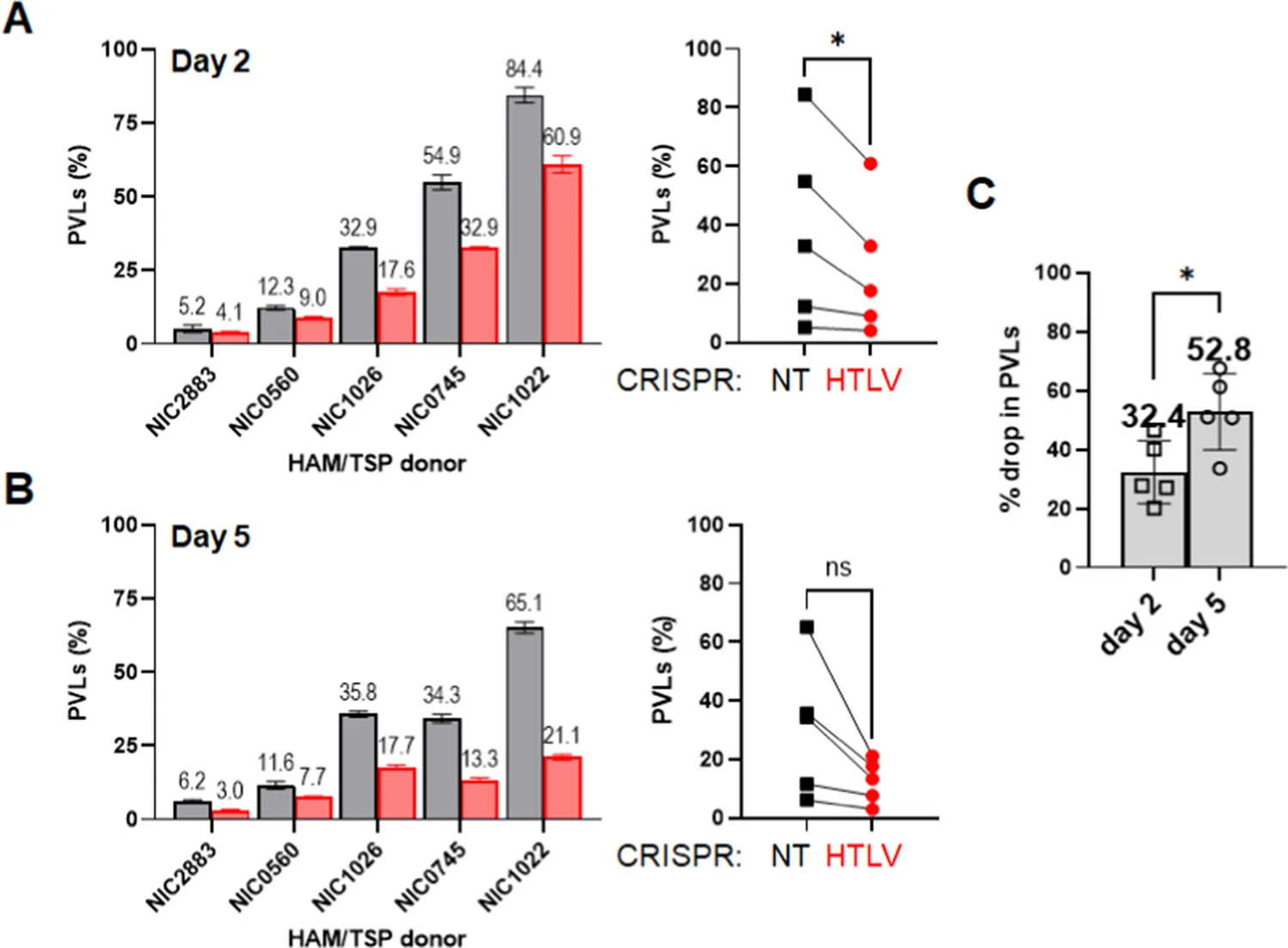
CRISPR treatment reduces proviral loads in PBMCs from HAM/TSP patients. qPCR quantification of proviral loads (PVLs) on day 2 **A** and 5 **B** post-CRISPR treatment. Grey bars/black squares represent cells treated with Cas9/gRNA complexes containing non-targeting gRNA (NT), and red bars/red circles represent cells treated with HTLV-1-env + 3’LTR specific gRNAs (HTLV). C) Plot showing average % drop in PVLs in CRISPR-HTLV-treated versus CRISPR-NT-treated cells. Paired t test, * *p* < 0.05, ns *p* = 0.072

Next, we performed qPCR analysis of the same genomic DNA to assess whether the presence of a 2613-bp deletion and corresponding reduction in the intensity of full-length amplicons observed in CRISPR-HTLV-treated cells relative to CRISPR-NT-treated cells (Fig. 1B), translates into a reduction in PVLs, the primary objective of the study. Indeed, CRISPR-HTLV treatment resulted in a statistically significant ~ 30% reduction in PVLs relative to control (CRISPR-NT) on day 2 post-treatment (Fig. 2A). Interestingly, we observed that PVLs declined further by day 5 in all donors except NIC1026, reaching > 50% relative to controls (Figs. 2B and C). Regarding donors with lower PVLs (NIC0667, NIC3024, NIC1306, and NIC1284), we also observed a reduction in PVLs, although less pronounced (Supplementary Fig. 3D). Lastly, there was no reduction of PVLs for donor NIC1306, for whom we could not verify env-3’LTR excision.

## Discussion

The work presented here is, to our knowledge, the first proof-of-concept study demonstrating that CRISPR technology can be used to excise the HTLV-1 genome and reduce PVLs in PBMCs from HAM/TSP patients. While the CRISPR platform has been extensively used to target HIV-1, only four reviews (Panfil et al. 2020; Scott and Morris 2021; Wilkie and Panfil 2022; Domingues et al. 2024) and two research papers (Nakagawa et al. 2018; Hashikura et al. 2025) have covered a CRISPR-based approach for HTLV-1. These papers primarily focus on the use of CRISPR for ATL, overlooking the effect of treatment for HAM/TSP. Nakagawa et al. showed that CRISPR-mediated disruption of the viral gene HBZ expression in the ATL cell line KK1 resulted in a time-dependent decrease in cell viability and proliferation (Nakagawa et al. 2018). Similarly, Hashikura et al. showed that another ATL cell line, ST-1, transfected with a CRISPR targeting HBZ exhibited significantly reduced proliferation (Hashikura et al. 2025). There are two additional research papers using gene-editing platforms other than CRISPR-Cas9 to target HTLV-1, again in ATL cell lines. Tanaka et al. showed that treatment of various HTLV-1-transformed and patient-derived ATL cell lines with ZFNs (zinc finger nucleases) targeting viral LTRs reduced proliferation in vitro and decreased tumor volume after transplantation into immunodeficient mice (Tanaka et al. 2013). Finally, Rojo-Romanos et al. developed an engineered Cre-derived site-specific recombinase targeting the conserved loxP-like sequence (loxHTLV) present in the long terminal repeats to excise the HTLV-1 proviral genome from the ATL cell line SP (Rojo-Romanos et al. 2023).

Our validated CRISPR-HTLV strategy involves excising the env-3’LTR segment of the HTLV-1 genome, which contains the pX region encoding several accessory genes, including tax, rex, p12, p21, p30, p13, and HBZ (Matsuoka and Jeang 2007). Two of these genes, tax and HBZ, have been demonstrated to play critical roles in reservoir maintenance and immune dysregulation in HAM/TSP (Enose-Akahata et al. 2017). Increased expression of the highly immunogenic Tax induces expression of various cellular genes, such as IL-2 (Siekevitz et al. 1987) and IL-15 (Azimi et al. 1998), which directly contribute to lymphocyte activation and immunopathogenesis in HAM/TSP patients (Jacobson et al. 1990; Azimi et al. 2001). On the other hand, low immunogenic and tolerated during immune surveillance HBZ (Suemori et al. 2009), markedly contributes to the proliferation of infected T cells (Zhao and Matsuoka 2012). Furthermore, Saito et al. reported that HBZ mRNA load is significantly correlated with HTLV-1 proviral load in HAM/TSP patients and with two independent clinical parameters reflecting disease: CSF neopterin concentrations (a marker of neuroinflammation) and motor dysfunction scores (the Osame Motor Disability Score) (Saito et al. 2009). Additionally, using HTLV-1 DNA-capture-seq, Katsuya et al. showed that almost all defective proviral genomes present in PBMCs from HTLV-1-positive people, regardless of their clinical status, were 5’-defective (loss of the 5’-LTR and Tax expression), accounting for ~ 15% of all proviruses, whereas 3’-defective sequences were very rare, at > 0.02% (Katsuya et al. 2019). This bias can be explained again by the critical role of the HBZ gene, encoded in the 3’-portion of the HTLV-1 genome (pX region) and expressed as an antisense transcript from the 3' LTR, in the maintenance of the pool of infected cells. Therefore, we anticipate that our CRISPR-HTLV strategy, excising the pX region to eliminate Tax and HBZ expression, will alleviate their immunopathogenic effects and yield optimal therapeutic outcomes for HAM/TSP patients.

### Study limitations

This is a preliminary proof-of-concept study using PBMCs from only ten HAM/TSP donors. Because our CRISPR delivery method, electroporation of Cas9/gRNA RNPs, requires highly viable cells (> 90%), we could not use freshly thawed cells. If transfected after thawing, the cells underwent massive cell death, even after a 1-day rest period. The protocol we established and followed (activating for 3 days and then culturing PBMCs for an additional 2–3 days) enabled us to obtain a suitably viable cell population, but at the expense of nonspecific lymphocyte expansion and changes in the CD4 +/CD8 + T-cell ratios. We did not develop a PCR assay or sequence the 5' LTR-env portion of the HTLV-1 genome; we focused only on the env-3' LTR region. Nevertheless, our 3’LTR-2034rev gRNA targets the R region of HTLV-1 LTR, which is also present in 5’LTR. Because of that, there are two more possible CRISPR excision events: 5’LTR-3’LTR and 5’LTR-env, which were not investigated here. Thus, it is important to remember that the reduction in PVLs observed with CRISPR-HTLV treatment can result from all three excision events, not only the env-3’LTR excision event demonstrated here. Moreover, due to funding constraints, we performed only basic off-target analysis. In the future, we plan to conduct more comprehensive, unbiased genome-wide off-target analyses using methods such as GUIDE-seq and WGS. Owing to the scarcity and restricted volume of available HAM/TSP clinical samples, the experimental protocol was confined to genomic DNA analysis to establish definitive proof of CRISPR excision. Consequently, we were unable to perform downstream functional readouts, such as evaluating HTLV-1 viral transcript expression via RT-qPCR, quantifying inflammatory cytokines (e.g., IFN-γ or TNF-α), or profiling T-cell activation markers. Characterizing these functional and immunological consequences remains a vital priority to fully elucidate the translational significance of this gene-editing approach. Our future work will focus on functional validation, utilizing immune cell-neuronal co-culture models to systematically evaluate whether CRISPR-mediated proviral reduction can successfully mitigate or prevent HTLV-1-associated, immune-mediated neuronal injury. In conclusion, despite the noted limitations, our CRISPR-HTLV approach achieved effective HTLV-1 PVL reduction, providing a foundation for future development of therapeutic approaches that may improve treatment options for people living with HAM/TSP and other HTLV-1-related diseases.

## Materials and methods

### Ethics statement

Peripheral blood samples from HAM/TSP patients were collected under protocol #98N0047 approved by the National Institutes of Health IRB #10, IRB Registration: IRB00011862 and the National Institute of Neurological Disorders and Stroke (NINDS) Scientific Review Committee. Written informed consent was obtained from subjects prior to study inclusion in accordance with the Declaration of Helsinki.

### Cell culture

PBMCs from HAM/TSP patients (Supplementary Table 1) were thawed and cultured in RPMI medium supplemented with 10% Fetal Bovine Serum and gentamycin (50ug/ml). After 24 h, cells were induced with ImmunoCult™ Human CD3/CD28 T Cell Activator (STEMCELL Technologies, #10991) for 3 days. Next, cells were counted, replated at a density of 10^6/ml in growth medium supplemented with recombinant human IL-2 at 10 ng/ml (PeproTech, #200–02-250UG), and cultured for an additional 2–3 days.

### Design and selection of candidate CRISPR gRNAs

To design CRISPR gRNAs, we used CRISPOR (http://crispor.tefor.net/) (Concordet and Haeussler 2018). The env-3’LTR sequence of NIC0071 donor was screened for possible 20-nucleotide-long gRNA protospacer regions followed by SpCas9-specific NGG PAM, using the GRCh38/hg38 + SNPs: dbSNP148 Kaviar reference human genome. Next, a set of 4 candidate gRNAs, which showed the highest on-target and the lowest nominated off-target activity (Supplementary Tables 2 and 3), was selected for further evaluation in PBMCs from the same donor (NIC0071).

### RNP-CRISPR electroporation

Synthetic guide RNAs were complexed with recombinant SpCas9 by adding 2.2 μl of Alt-R™ S.p. Cas9 Nuclease V3 (10 mg/ml, Integrated DNA Technologies Inc., cat#1081059) to 6 μl of synthetic gRNA (60 μM Alt-R CRISPR-Cas9 gRNA reconstituted in Nuclease Free Duplex Buffer, Integrated DNA Technologies Inc., cat#11–01–03–01). SpCas9 complexes containing gRNA specific to the mouse SLC22a17 gene, which does not target human and HTLV-1 genomes, were used as a non-targeting control (Supplementary Table 4). The mixtures were incubated at room temperature for 20 min, then mixed with PBMCs suspended in 92 μl of P3 electroporation buffer. Cells were then gently mixed, transferred into 100 µl electroporation cuvettes (Lonza Walkersville Inc., Walkersville, MD, USA, cat# V4XP-3024), and electroporated using a 4D-Nucleofector X unit with EH-100 settings (Seki and Rutz 2018). Immediately after electroporation, 500 μL of prewarmed growth medium was added to each cuvette, and the electroporation cuvettes were placed in the tissue culture incubator for 5 min to allow cell recovery. Subsequently, the cells were transferred to 6-well plates containing prewarmed growth medium (2 ml per well per condition), mixed gently, and returned to the tissue culture incubator.

### DNA extraction and PCRs

After 2 and 5 days, genomic DNA was extracted from the cells using the NucleoSpin Tissue kit (Macherey–Nagel, cat. #740952.250) according to the manufacturer’s protocol and subjected to PCR with primers flanking the CRISPR-targeted env-3’LTR region of the HTLV-1 genome (Supplementary Table 4). Platinum™ SuperFi II PCR Master Mix (Thermo Fisher Scientific, cat. #12368010) was used for PCR amplification, with 200 ng of genomic DNA per reaction and a 3-min extension time. For nominated off-target sites PCRs, a shorter 30-s extension time was used. The site-specific amplicons were then extracted from the gel using a PCR and Gel Extraction Kit (Macherey–Nagel, cat. #740609.250) and sent for Sanger sequencing (Azenta Life Sciences, USA).

### Sanger sequence analysis

For sequence alignments and phylogenetic tree analysis, Sanger FASTA files were analyzed with Clustal Omega Multiple Sequence Alignment (MSA) software (https://www.ebi.ac.uk/jdispatcher/msa/clustalo) (Madeira et al. 2024). For verification of truncated CRISPR-cleaved/end-joined amplicons Sanger sequencing chromatograms (.ab files) were analyzed using Snap Gene Viewer software (www.snapgene.com). For the detection of CRISPR-induced InDel mutations, Sanger chromatograms (.ab files) were analyzed with ICE software (Inference of CRISPR Edits; https://ice.editco.bio/) (Conant et al. 2022).

### Proviral loads

Genomic DNA was used in qPCRs (50 ng/well) using TaqMan primer/probe sets specific to HTLV-1 tax gene and human hTert as the genome copy number reference (Supplementary Table 4). The % PVLs were calculated as follows: HTLV-1 tax/hTert ratio × 2 (two copies of hTert in diploid cell) × 100. The % PVL drop was calculated with the formula: 100—(CRISPR-HTLV PVL/CRISPR-NT PVL × 100).

### Statistical analysis

All values presented on the graphs are given a mean ± SEM. Paired Student’s t-test was used to analyze the statistical significance. Statistical analyses were performed using GraphPad Prism 10.1.2 and are indicated in the figure legends.

## Supplementary Information

Below is the link to the electronic supplementary material.

Supplementary file1 (DOCX 4767 KB)

## Data Availability

The original contributions presented in this study are included in the article and **Supplementary Materials**. Further inquiries can be directed to the corresponding author.
