# Supplementary material for: CRISPR-mediated excision of HTLV-1 reduces proviral loads in PBMCs from HAM/TSP patients
Source: J Neurovirol. 2026 Aug 29;32(5):41. doi: 10.1007/s13365-026-01333-7 (PMC13526106; doi:10.1007/s13365-026-01333-7)
Supplement: Supplementary file 1 [file 13365_2026_1333_MOESM1_ESM.docx]

**Supplementary Materials**.

**Supplementary Table 1: HAM/TSP donor PBMCs.**

| Sample# | Donor ID# | Cells/ml | date | Gender |
| --- | --- | --- | --- | --- |
| 1 | NIC0071 | 3.0E+07 | 8/11/2015 | Male |
| 2 | NC0560 | 2.0E+07 | 10/9/2014 | Female |
| 3 | NIC0667 | 4.0E+07 | 5/31/2019 | Female |
| 4 | NIC0745 | 5.0E+07 | 2/23/2009 | Male |
| 5 | NIC1022 | 2.5E+07 | 7/17/2019 | Female |
| 6 | NIC1026 | 5.0E+07 | 3/9/2012 | Female |
| 7 | NIC1284 | 2.0E+07 | 1/23/2014 | Female |
| 8 | NIC1306 | 5.5E+07 | 4/24/2014 | Male |
| 9 | NIC2883 | 2.0E+07 | 7/12/2018 | Female |
| 10 | NIC3024 | 4.0E+07 | 12/13/2019 | Male |

**Supplementary Table 2: CRISPR gRNAs targeting HTLV-1 NIC0071 isolate sequence.**

| #guideId | targetSeq | mitSpecScore | cfdSpecScore | offtargetCount |
| --- | --- | --- | --- | --- |
| **312rev** | **AGACCGCCACCGGTACCGCTCGG** | **100** | **99** | **7** |
| 791rev | ATGGGGGTATTTGACGCGCGAGG | 99 | 99 | 9 |
| 1993rev | GCGGCAGAACGCGACTCAACCGG | 99 | 100 | 9 |
| **2034rev** | **CTAGACGGCGGACGCAGTTCAGG** | **99** | **98** | **8** |
| 789rev | GGGGGTATTTGACGCGCGAGGGG | 98 | 99 | 20 |
| 790rev | TGGGGGTATTTGACGCGCGAGGG | 98 | 99 | 23 |
| 1984rev | CGCGACTCAACCGGCGTGGATGG | 98 | 99 | 7 |
| 1988rev | AGAACGCGACTCAACCGGCGTGG | 98 | 99 | 8 |
| 150rev | GGGCTGGAAGCGCTAACGATGGG | 97 | 99 | 26 |
| 323forw | CCGCTCCCGCCGAGCGGTACCGG | 97 | 99 | 22 |

**Supplementary Table 3: Nominated off-target sites for HTLV-1 gRNAs env-312rev and 3’LTR-2034rev.**

| Off-target site name | offtargetSeq | mismatchPos | Mismatch# | chrom  location | strand | locusDesc |
| --- | --- | --- | --- | --- | --- | --- |
| 312rev-OFF1-Chr1 | AGACCACCACAGGTAAAGCTAGG | .....*....*....**... | 4 | chr1:  62410291-62410313 | + | intergenic:KANK4-USP1 |
| 312rev-OFF2-Chr5 | AGAGCGCCACTGGTGCCTCTAGG | ...*......*...*..*.. | 4 | chr5:  51173198-51173220 | + | intergenic:RNU6-1296P-CTD-2314G24.2 |
| 312rev-OFF3-Chr6 | GGACCCCAACCGGTACCCCTCGG | *....*.*.........*.. | 4 | chr6:  52237137-  52237159 | - | exon:IL17F |
| 312rev-OFF4-Chr7 | AGACCACCACCAGTTCTGCTAGG | .....*.....*..*.*... | 4 | chr7:  43873767-43873789 | + | intergenic:MRPS24-URGCP |
| 312rev-OFF5-Chr16a | AGGCCGCCGCGGGTGCCGCTGGG | ..*.....*.*...*..... | 4 | chr16:  25257609-25257631 | + | exon:ZKSCAN2 |
| 312rev-OFF6-Chr16b | AGACCGACACTGGTAGCCCTGGG | ......*...*....*.*.. | 4 | chr16:  23792032-23792054 | - | intergenic:CHP2-PRKCB |
| 312rev-OFF7-Chr17 | ACACCGCCACCGAGACCGGTAGG | .*..........**....*. | 4 | chr17:  16535650-16535672 | + | intergenic:LRRC75A-ZNF287 |
| 2034rev-OFF1-Chr1 | CTAGAAGGCTGCCCCAGTTCAGG | .....*...*.*.*...... | 4 | chr1:  9347845-9347867 | + | intron:SPSB1 |
| 2034rev-OFF2-Chr2 | CTAAACGGAGAACACAGTTCTGG | ...*....*.*..*...... | 4 | chr2:  46096341-46096363 | + | intron:PRKCE |
| 2034rev-OFF3-Chr6 | CTGGAAGGATGACGCAGTTCTGG | ..*..*..**.......... | 4 | chr6:  158543296-158543318 | + | intron:TMEM181 |
| 2034rev-OFF4-Chr10 | CTGGACCGCGGAGGCAGTGCAGG | ..*...*.....*.....*. | 4 | chr10:  97914641-97914663 | + | intron:CRTAC1 |
| 2034rev-OFF5-Chr11 | GTAGACGGCGGCAGCAGCTCCGG | *..........**....*.. | 4 | chr11:  73264540-73264562 | - | exon:P2RY6 |
| 2034rev-OFF6-Chr18 | CCAGAAGGCGGAAGCAGCTCAGG | .*...*......*....*.. | 4 | chr18:  8967036-8967058 | + | intergenic:MTCL1-NDUFV2/RP11-21J18.1 |
| 2034rev-OFF7-Chr20 | TCAGACGGAGGACGCAGCTCAGG | **......*........*.. | 4 | chr20:  63785722-  63785744 | + | intron:ZBTB46 |
| 2034rev-OFF8-ChrX | CAAGATGGCTGAGGCAGTTCTGG | .*...*...*..*....... | 4 | chrX:  27866672-27866694 | + | intergenic:MAGEB10-DCAF8L1 |

**Supplementary Table 4: gRNAs and primers.**

| Name | Sequence 5’-3’ |
| --- | --- |
| gRNAs: | |
| HTLV-1 env-312rev | AGACCGCCACCGGTACCGCT**CGG** |
| HTLV-1 env-791rev | ATGGGGGTATTTGACGCGCG**AGG** |
| HTLV-1 3’LTR-1993rev | GCGGCAGAACGCGACTCAAC**CGG** |
| HTLV-1 3’LTR-2034rev | CTAGACGGCGGACGCAGTTC**AGG** |
| mouse SLC22a17-218forw (non-targeting control gRNA) | GCCCGTGCTTTTCGTGGCGC**TGG** |
| On-target PCR primers | |
| env-Forward-set1 | ATGACCATGAGCCCCAAA |
| 3’LTR-Reverse-set-1 | GATCAAGGCCTCGTCTGTTC |
| env-Forward-set2 | GAGCCCTCTATACCATGGAAATC |
| 3’LTR-Reverse-set-2 | TAACGGCGCAGAACAGAAA |
| env-Forward-set3 | CACTTGGCACGTCCTATACTC |
| qPCR TaqMan primers/probe | |
| tax-Forward | CGGATACCCAGTCTACGTGTT |
| tax-Reverse | CAGTAGGGCGTGACGATGTA |
| tax-probe-FAM | CTGTGTACAAGGCGACTGGTGCC |
| hTert-Forward | TGGAGCAAGTTGCAAAGCAT |
| hTert-Reverse | CAGAGCCTTGCACAGAATCC |
| hTert-probe-HEX | CCGGCCTCAGCATGCGCCTG |
| Off-target PCR primers | |
| 312rev-OFF1-Forward* | CCCAAGAAGTTGATGGTGACA |
| 312rev-OFF1-Reverse** | TCTTCAAGCCAAGGGCAAA |
| 312rev-OFF2-Forward* | CCACCAGCTATGCCTTTCA |
| 312rev-OFF2-Reverse** | TCGCATTGACCTTCTTTAGGAT |
| 312rev-OFF3-Forward* | ACCTTCTCCAACTGGAAAGAAA |
| 312rev-OFF3-Reverse** | TGAACACTCAGTGCTGTGG |
| 312rev-OFF4-Forward* | CAGGATAGGTTTATGGCCAAATTA |
| 312rev-OFF4-Reverse** | CATGTCAGATCAGGATTCATCTTT |
| 312rev-OFF5-Forward* | GGGTGTGTCCGCTACTC |
| 312rev-OFF5-Reverse** | TCCGGGCGGAACAGTTT |
| 312rev-OFF6-Forward* | TGACAGTTAGCTGCCCAAATC |
| 312rev-OFF6-Reverse** | TTCTTGTGCCCTCTGAGGTA |
| 312rev-OFF7-Forward* | TTATTGCCAGTGTGTGGGT |
| 312rev-OFF7-Reverse** | CGCAGTGTTCTTGGGAAATG |
| 2034rev-OFF1-Forward* | CCAGCATCTTCATCCTCATGT |
| 2034rev-OFF1-Reverse** | CGTCAGAGCGAGATTCTGTTTA |
| 2034rev-OFF2-Forward* | TTCTCAGTGTTCCATGGGAATTA |
| 2034rev-OFF2-Reverse** | CCCTATCTCCAAATGCCATC |
| 2034rev-OFF3-Forward* | CCTGGCCTCAGAGTATGTTTATT |
| 2034rev-OFF3-Reverse** | CGATACATCCCTGTTGCTTGA |
| 2034rev-OFF4-Forward* | GGCTGTAATGCACAGGGTAG |
| 2034rev-OFF4-Reverse** | CTTGAAGCCGGGACAGTG |
| 2034rev-OFF5-Forward* | CAGCCTCTTCCAGACATCAC |
| 2034rev-OFF5-Reverse** | AGGCCTCCCTGTATCCC |
| 2034rev-OFF6-Forward* | GAAACGGTATGGCAATTCCTTAAA |
| 2034rev-OFF6-Reverse** | ACTATGTTGTAACCTGTGTCTGAA |
| 2034rev-OFF7-Forward* | GGGCCTTTGCTTCTATAAATGG |
| 2034rev-OFF7-Reverse** | TTCCGAGTTGCTAAGATGAGG |
| 2034rev-OFF8-Forward* | AACACAACTCAAACTGAACTGA |
| 2034rev-OFF8-Reverse** | GTGGACAAGTGACATAAATGGA |

Partial Illumina Adapter Sequences were added to the 5’-end of all off-target primers:

*For the forward sequencing read: 5’-ACACTCTTTCCCTACACGACGCTCTTCCGATCT-3’

**For the reverse sequencing read: 5’-GACTGGAGTTCAGACGTGTGCTCTTCCGATCT-3’

**Supplementary Figure 1. HTLV-1 3’LTR sequences from our HAM/TSP cohort cluster with HTLV-1a-TC (subtype a (cosmopolitan) subgroup A (Transcontinental)) LTRs.** A phylogenetic tree showing hierarchical clustering of HTLV-1 LTR sequences obtained from our cohort of ten HAM/TSP patients and LTR sequences extracted from the public HTLV-1 molecular epidemiology database (Araujo 2012). All LTR sequences in the database derive from subtype a (cosmopolitan). Ten of LTR sequences from each: subgroup A (Transcontinental, dark blue), B (Japanese, green) and D (North African, violet) were randomly selected for sequence alignment together with all available sequences for subgroup C (West African, 4 LTR sequences, red) and subgroup E (Peruvian, 1 LTR sequence, black).

**Supplementary Figure 2. CRISPR-mediated editing of env-3’LTR region of HTLV-1 genome in PBMCs from HAM/TSP donor NIC0071 (HTLV-1a-TC).** **A)** Agarose gel picture of PCR genotyping of HTLV-1 env-3’LTR region from PBMCs of HAM/TSP donor NIC0071 2 days after treatment with non-targeting (NT) or HTLV-1 env and/or 3’LTR targeting (HTLV) Cas9/gRNA complexes. Black arrow points to 3189bp full-length env-3’LTR amplicon (Suppl.Table 4., PCR primer set 1). Blue arrows mark amplicons that were sent for Sanger sequencing verification. B) Sequence alignments obtained from ICE (inference of CRISPR edits) analysis of Sanger sequences of full-length amplicons from A). The sequences and frequencies of different InDel mutations detected at the gRNA target sites are shown. The total number of InDel mutations detected for each gRNA is shown on the left. **C)** Sanger sequencing chromatograms of truncated amplicons from A). gRNA env-312rev target site is highlighted in green, 3’LTR-1993rev target site in pink, 3’LTR-2034rev target site in blue, PAM sequences in red, and ambiguous/mismatched nucleotides in yellow. The site of CRISPR-excision “scars”, i.e., end-joined env-to-3’LTR sequences, are depicted by red arrows and a dashed red line (break point).

**Supplementary Figure 3. CRISPR-mediated excision of env-3’LTR region of HTLV-1 genome in PBMCs from HAM/TSP patients with low PVLs.** **A)** Agarose gel picture of PCR genotyping of HTLV-1 env-3’LTR region from five HAM/TSP patient PBMCs 2 days after treatment with non-targeting (NT) or HTLV-1-env+3’LTR targeting (HTLV) Cas9/gRNA complexes. Black arrow points to 3112bp full-length env-3’LTR amplicon (Suppl.Table 4., PCR primer set 2). Red arrow points to truncated 498bp amplicon resulting from CRISPR-mediated excision of 2613bp segment of HTLV-1 genome. **B)** Agarose gel picture of semi-nested PCR performed using as a template 3ul of PCR reactions shown in A) (Suppl.Table 4., env-Forward-set3 + 3’LTR-Reverse-set2). **C)** Sanger sequencing chromatograms of truncated amplicons from B) (6-8). gRNA env-312rev target site is highlighted in green, 3’LTR-2034rev target site in blue, PAM sequences in red, and ambiguous/mismatched nucleotides in yellow. The site of CRISPR-excision “scars”, i.e., end-joined env-to-3’LTR sequences, are depicted by red arrows and a dashed red line (break point). NS denotes non-specific, uninterpretable sequences (PCR artefact). **D)** qPCR quantification of proviral loads (PVLs) on day 2 (left) and 5 (right) post-CRISPR treatment. Grey bars represent cells treated with Cas9/gRNA complexes containing non-targeting gRNA (NT), and red bars represent cells treated with HTLV-1-env+3’LTR specific gRNAs (HTLV).

**Supplementary Figure 4. Off-target analysis for HTLV-1 gRNA env-312rev. A)** Agarose gel pictures of PCR genotyping of nominated off-target sites in the human genome for gRNA env-312rev. Genomic DNAs collected from three HAM/TSP patient PBMCs (NIC0745, lines 1-2, NIC1022, lines 3-4, and NIC1026, lines 5-6) 2 days after treatment with non-targeting (NT) or HTLV-1-env+3’LTR targeting (HTLV) Cas9/gRNA complexes were used as a template (same as used in Figure 1 and Figure 2). **B)** Example of Sanger sequencing chromatogram alignments obtained from ICE (inference of CRISPR edits) analysis of Sanger sequences of amplicons from A) for HAM/TSP donor NIC0745. No InDel mutations were detected in any of the three donors.

**Supplementary Figure 5. Off-target analysis for HTLV-1 gRNA 3’LTR-2034rev. A)** Agarose gel pictures of PCR genotyping of nominated off-target sites in the human genome for gRNA 3’LTR-2034rev. Genomic DNAs collected from three HAM/TSP patient PBMCs (NIC0745, lines 1-2, NIC1022, lines 3-4, and NIC1026, lines 5-6) 2 days after treatment with non-targeting (NT) or HTLV-1-env+3’LTR targeting (HTLV) Cas9/gRNA complexes were used as a template (same as used in Figure 1 and Figure 2). **B)** Example of Sanger sequencing chromatogram alignments obtained from ICE (inference of CRISPR edits) analysis of Sanger sequences of amplicons from A) for HAM/TSP donor NIC0745. No InDel mutations were detected in any of the three donors.
